# Screening for Clonal Hematopoiesis for Mitigating the Risk of Hematopoietic Neoplasms after PRRT

**DOI:** 10.1055/s-0043-1764308

**Published:** 2023-04-28

**Authors:** Piyush Chandra, Kishore Kumar

**Affiliations:** 1 Department of Nuclear Medicine, Zydus Hospitals, Vadodara, Gujarat, India; 2 Department of Hematology, MIOT International, Chennai, Tamil Nadu, India

^177^ Lu-DOTATATE, a peptide receptor-based radionuclide therapy (PRRT), is one of latest treatment options for patients with progressive gastro-entero-pancreatic neuroendocrine tumors (NETs) and leads to significantly better disease-free survival. 1 Although rare, an ominous adverse effect seen with PRRT is development of myelodysplastic syndrome (MDS) or acute myeloid leukemia (AML). Recently, a global multicenter study done by Vigne et al using the World Health Organization pharmacovigilance database VigiBase including 1,674 cases, showed 0.91 and 0.31% incidence of MDS and AML, respectively. These adverse events were associated with treatment discontinuation in all affected patients, and more importantly approximately one-third of these cases eventually had fatal outcomes. 2

Based on accumulated clinical data over the past decade and a half, incidence of PRRT-related myeloid neoplasms (t-MN) has been reported in 0.2 to 5.4% of the patients. 3 4 5 6 Long-term follow-up data from Erasmus Medical Centre, including 1,214 patients showed MDS incidence at 1.5% after a median follow-up of 28 months and acute leukemia at 0.7% after a median follow-up of 55 months after first therapy. 7 The final results of the NETTER-1 study group showed t-MN risk at 1.2% post 5 years of the last patient is randomized. 8 A slightly higher percentage of patients experienced t-MN after PRRT in two other studies, both of which combined PRRT with prior or concomitant chemotherapy. Of note, a much higher rate of t-MN (20%) was reported by Brieau et al in a limited series of 20 nonresectable NETs treated with ^177^ Lu-PPRT after heavy pretreatment with chemotherapy. 9 Another study done by Goncalves et al from the Peter MacCallum Cancer center including 521 patients over a 12-year period showed 4.8% incidence of t-MN. 10 Twenty-five percent of these patients had received prior chemotherapy with carboplatin/etoposide and 88% received concomitant radiosensitizing chemotherapy such as 5FU or capecitabine. The median overall survival (OS) after diagnosis of t-MN was shown to be mere 13 months. Although the novel approach of PRRT with combined chemotherapy may potentially offer better tumor control, it may also slightly augment the risk of t-MN.

The quest for identifying predictive biomarkers for post-t-MN continues. Unlike nephrotoxicity which is considered dose-dependent side effect of PRRT, occurrence of long-term hematological toxicity is difficult to predict based on marrow dosimetry alone. 11 A study done by Brieau et al showed two prognostic factors for the development of t-MN identified in this study: (1) early grade 3 to 4 hematological toxicity after PRRT and (2) higher number of chemotherapy cycles before PRRT. Similarly, post-PRRT thrombocytopenia was significantly related to the development of secondary MDS or AML in a previous study. 5 Hence, close monitoring should be recommended in patients experiencing early hematological toxicity after PRRT.

A novel strategy to mitigate the risk of t-MN appears to be pretreatment identification of clonal hematopoiesis (CH) in patients at risk for t-MN. The acquisition of somatic mutations detected in the blood leading to the clonal expansion of mutated hematopoietic cells is referred to as CH. CH is commonly detected in healthy individuals; however, it is also associated with risk of hematologic disease. CH mutations generally occur at low frequencies in genes implicated in myeloid neoplasms such as DNMT3A, TET2, ASXL1, and TP53. In a large study analyzing next-generation sequencing (NGS) data from approximately 8,810 patients, CH was identified in approximately 25% of the non-hematopoietic cancer patients at baseline and shown to be associated with increasing age, prior chemotherapy, radiation therapy, and tobacco consumption. 12

Recently published data by Singh et al points toward CH being one of the reasons for the development of t-MN after PRRT. They evaluated pre- and post-PRRT blood samples of 13 patients with NET analyzing the genomic deoxyribonucleic acid (DNA) using a 100-gene panel. A variant allele frequency cutoff of 1% was used to call CH. Sixty-two percent of the patients had CH at baseline (this is more than 25% incidence which is seen at baseline in other solid tumors). In 64% of patients with documented cytopenias post-PRRT, clonal expansion of mutant DNA damage response genes ( *TP53* , *CHEK2* , and *PPM1D* ) were seen, most commonly in PPM1D and TP53 genes. 13 The data from the Peter MacCallum group, using NGS myeloid amplicon gene panel, also showed mutations in TP53 being the most frequent one in patients developing t-MN. 10 However, before CH detection can be employed in routine clinical protocols, certain factors about standardization/definition of CH need to be addressed. Major factors include sequencing depth, the set of genes sequenced, and the minimum percentage of blood cells with the mutation (i.e., variant allele fraction [VAF]) used for CH calling. Currently, a cutoff for CH as a somatic mutation in the peripheral blood at a VAF of 2% or greater has been suggested. 14

The use of PRRT (now Food and Drug Administration approved) for patients with mid-gut NET is only going to grow. Real-world data may witness a probably higher incidence of side effects compared with those done in industry-driven trials performed in a controlled environment. The ominous development of t-MN, although seen in very few patients (1–5%), is very concerning, given limited treatment options. Emerging data points toward a possibility of individual susceptibility to develop myeloid neoplasms after PRRT exposure. Dismal survival in t-MN calls for various risk-mitigating strategies such as avoidance of avoiding alkylating chemotherapy in patients with low grade NET (associated with long OS), close monitoring of blood counts post-PRRT, and a novel approach of pretreatment and longitudinal screening of CH by identifying premalignant clones with mutant DNA damage response genes (PPM1D and TP53). Personalized PRRT is indeed the way forward for improving overall outcomes in NET patients.
